# Analytical treatment of the fractional Zakharov–Kuznetsov equation via the generalized integral residual power series method

**DOI:** 10.1038/s41598-025-09102-y

**Published:** 2025-07-15

**Authors:** Samy A. Abdelhafeez, Anas A. M. Arafa, Yousef H. Zahran, Moutaz Ramadan, Ibrahim S. I. Osman

**Affiliations:** 1 Department of Mathematics and Computer Science, Faculty of Science, Port Said University, Port Said, Egypt; 2 Department of Mathematics, College of Science, Qassim University, P. O. Box 6644, 51452 Buraydah, Saudi Arabia; 3 Department of Mathematics, College of Sciences and Humanities, Prince Sattam Bin Abdulaziz University, Alkharj, Saudi Arabia; 4 Physics and Mathematics Department, Faculty of Engineering, Port Said University, Port Said, Egypt

**Keywords:** Fractional derivatives, New general integral transform, Residual power series method, Zakharov–Kuznetsov equation, Numerical results, Applied mathematics, Computational science

## Abstract

This study presents a generalized integral residual power series method (GIRPSM) for finding semi-analytical solutions to the nonlinear fractional Zakharov–Kuznetsov equation (FZKE). This method combines the residual power series method with a new general integral transform to improve accuracy and convergence. The effectiveness of this method is demonstrated by the robustness of the numerical results. The results demonstrate that GIRPSM is highly accurate and reliable in solving nonlinear fractional partial differential equations, including those modeling plasma wave propagation.

## Introduction

Differential equations whether ordinary or partial are considered one of the great importance branches of natural science. Recently, at the end of the seventeenth century, the fractional differential equations (FDEs) have appeared. The FDEs are the general extension of calculus from integer order to arbitrary order. It has been demonstrated that fractional calculus’s primary benefit is its great ease of use in characterizing the memory and genetic characteristics of many phenomena; also, traditional calculus is merely a limited subset of fractional calculus. Liouville^1^, Riemann^2^, Caputo^3^, Miller and Ross^4^, Podlubny^5^, and numerous more pioneers worked together to lay the mathematical groundwork for fractional degree derivatives.

Fractional calculus theory has been used to chaos theory^6^, signal processing^7^, electrodynamics^8^, optics^9^, and biology^10,11^ and has been connected to real-world initiatives. The nature and behavior of nonlinear issues that occur in daily life are crucially described by the analytical and numerical solution of differential equations that result from the fractional order in the aforementioned phenomena^12–23^. Several methods are used to study the FZK equations such as the variational iteration method^24^, the homotopy perturbation method^25^, the fractional sub-equation method^26^, the residual power series method (RPSM)^27–31^, the Sumudu transform^32^, the Laplace–Adomian decomposition method^33^, the q-homotopy analysis transform method^34^, the modified auxiliary equation method^35^, the Shehu transform^36^, the optimal auxiliary function method^37–39^ and the non-polynomial spline method^40–43^.

The main objective of this paper is to develop and apply the GIRPSM to obtain approximate analytical solutions to the FZKE:

1 $$D_{t}^{\alpha } H + \left( {H^{n} } \right)_{x} + B\left( {H^{n} } \right)_{xxx} + B\left( {H^{n} } \right)_{xyy} = 0,$$

where B is dispersion coefficient, α is the order of the fractional derivative and $n$ is nonlinearity indexed positive integer number. The evolution term is represented by the first term, the wedge term by the second, and the dispersion term by the third and fourth terms taken together. Dispersion and nonlinearity are delicately balanced to produce isolated waves. The novelty of this work lies in the development and application of GIRPSM as a new semi-analytical technique for finding a new solution to the FZKE equation, a nonlinear fractional partial differential equation of great physical importance. The motivation behind proposing the GIRPSM method for solving the FZKE is the need for an efficient and accurate method to deal with the complexity of nonlinear fractional equations.

The advantages of this method are that it efficiently reduces the approximation error by correcting the residual at each step, allowing for highly accurate solutions even with low-degree truncation of series. The GIRPSM method handles nonlinear and fractional equations, making it versatile for a wide range of scientific and engineering problems. GIRPSM provides a clear solution in the form of a series rather than purely numerical data points, providing a better understanding of the behavior of the solution.

The physical application of the Fractional Zakharov–Kuznetsov Equation (FZKE) is focused on modeling nonlinear electromagnetic wave propagation in magnetized plasmas, particularly in systems with memory or nonlocal interactions. The incorporation of fractional derivatives enhances the model’s ability to capture realistic physical behaviors that cannot be represented using classical (integer-order) models.

This paper includes an expanded history of fractional calculus in Section “Preliminaries”. Section “Constructing the GIRPSM for the FZKE” describes the steps of GIRPSM for FZKE. The error norms are presented in Section “The error norms”. Numerical examples are presented in Section “Numerical Examples”. Section “Discussions” presents the discussions and conclusions presented in Section “Conclusion”.

## Preliminaries

We will go over a few definitions of fractional calculus in this section.

### Definition 1

(^5^) The Riemann–Liouville fractional integral of order $\alpha$ is given as:

$$\begin{aligned} J^{\alpha } g\left( y \right) & = \frac{1}{{{\Gamma }\left( \alpha \right)}}\mathop \smallint \limits_{0}^{x} \left( {y - t} \right)^{\alpha - 1} g\left( t \right)dt,\quad \alpha > 0,\quad y > 0, \\ J^{0} g\left( y \right) & = g\left( y \right), \\ \end{aligned}$$

### Definition 2

(^5^) The $\alpha^{{th{ }}}$ fractional derivative of order Caputo time of $H\left( {x,t} \right)$ is defined as:

$$D_{t}^{\alpha } { }H\left( {x,t} \right) = \left\{ {\begin{array}{*{20}l} {\frac{1}{{{\Gamma }\left( {m - \alpha } \right)}}\mathop \smallint \limits_{0}^{t} \left( {t - \zeta } \right)^{m - \alpha - 1} \frac{{\partial^{m} H\left( {x,\zeta } \right)}}{{\partial t^{m} }}d\zeta ,\quad m - 1 < \alpha < m,} \hfill \\ {\frac{{\partial^{m} H\left( {x,\zeta } \right)}}{{\partial t^{m} }},\quad \alpha = m \in N.} \hfill \\ \end{array} } \right.$$

### Definition 3

For t $\ge$ 0, $\varphi \left( s \right) \ne 0$ and q(s) are positive real function, the new general integral transform ${\text{\rm T}}$ of integrable function h(t) define by the form^44–47^:

$${\text{\rm T}}\left\{ {{\text{h}}\left( {{\text{x}},{\text{t}}} \right)} \right\} = {\text{H}}\left( {{\text{x}},{\text{s}}} \right) = \varphi \left( s \right)\mathop \smallint \limits_{0}^{\infty } h\left( {x,t} \right)e^{ - q\left( s \right)t} dt.$$

The following are some fundamental characteristics of the new general integral transform.:

(i) $${\text{\rm T}}[{ }D_{t}^{n\alpha } {\text{h}}\left( {{\text{x}},{\text{t}}} \right)] = q^{n\alpha } \left( s \right) {\text{H}}\left( {{\text{x}},{\text{s}}} \right) - \varphi \left( s \right)\mathop \sum \limits_{k = 0}^{n - 1} q^{{\left( {n - k} \right)\alpha - 1}} \left( s \right)D_{t}^{k\alpha } h\left( {x,0} \right),\quad n - 1 < \alpha \le n,{\text{n}} \in {\text{\rm Z}}^{ + } .$$

(ii) $${\text{\rm T}}\left[ {t^{\alpha } } \right] = \frac{\varphi \left( s \right)}{{q^{\alpha + 1} \left( s \right)}} {\Gamma }\left( {1 + \alpha } \right),\alpha > 0$$

(iii) $${\text{\rm T}}\left[ {\sin \left( {{\text{at}}} \right)} \right] = \frac{a \varphi \left( s \right)}{{q^{2} \left( s \right) + a^{2} }},$$

(iv) $${\text{\rm T}}\left[ {\cos \left( {{\text{at}}} \right)} \right] = \frac{a \varphi \left( s \right) q\left( s \right)}{{q^{2} \left( s \right) + a^{2} }},$$

(v) $${\text{\rm T}}\left[ {e^{at} } \right] = \frac{\varphi \left( s \right)}{{q\left( s \right) - a}}.$$

### Theorem 1

(^47^) Suppose that $H\left( {x,y,s} \right) = T\left( {h\left( {x,y,s} \right)} \right)$ has a fractional power series as:

$$H\left( {x,y,s} \right) = \varphi \left( s \right)\mathop \sum \limits_{n = 1}^{\infty } \frac{{f_{n} \left( {x,y} \right)}}{{q^{n\alpha + 1} \left( s \right)}},$$

then $$f_{n} \left( {x,y} \right) = D_{t}^{n\alpha } u\left( {x,y,0} \right)$$ where $$D_{t}^{n\alpha } \;{\text{is}}\;{\text{the}}\;{\text{product}}\;{\text{of}}\;D_{t}^{\alpha } \left( {n - times} \right)$$.

### Proof

See^47^.

## Constructing the GIRPSM for the FZKE

In this part, we demonstrate the FZKE as follows:

2 $$D_{t}^{\alpha } {\text{H}}\left( {{\text{x}},{\text{y}},{\text{t}}} \right) + D_{x} H^{n} \left( {{\text{x}},{\text{y}},{\text{t}}} \right) + {\text{B}}D_{x}^{3} H^{n} \left( {{\text{x}},{\text{y}},{\text{t}}} \right) + {\text{B}}D_{xyy} H^{n} \left( {{\text{x}},{\text{y}},{\text{t}}} \right) = 0,0 < \alpha \le 1.$$

The main idea of GIRPSM can be explained into the following steps:

*Step 1* Apply a new general integral transform to Eq. (2)

3 $$T\left[ { D_{t}^{\alpha } {\text{H}}\left( {{\text{x}},{\text{y}},{\text{t}}} \right)} \right] + {\text{ T}}\left[ {{ }\left\{ {D_{x} (T^{ - 1} H\left( {{\text{x}},{\text{y}},{\text{t}}} \right))^{n} } \right\}} \right] + {\text{BT}}\left[ {{ }\left\{ {{ }D_{x}^{3} (T^{ - 1} H\left( {{\text{x}},{\text{y}},{\text{t}}} \right))^{n} } \right\}} \right] + {\text{ BT}}\left[ {{ }\left\{ {D_{xyy} { }(T^{ - 1} H\left( {{\text{x}},{\text{y}},{\text{t}}} \right))^{n} } \right\}} \right] = 0.$$

Using definition 3 we get:

4 $$\begin{aligned} & q^{\alpha } \left( s \right) H\left( {x,y,s} \right) - \varphi \left( s \right)q^{\alpha - 1} \left( s \right) H\left( {x,y,0} \right) + {\text{ T}}\left[ {{ }\left\{ {D_{x} (T^{ - 1} H\left( {{\text{x}},{\text{y}},{\text{t}}} \right))^{n} } \right\}} \right] \\ & \quad + {\text{BT}}\left[ {{ }\left\{ {{ }D_{x}^{3} (T^{ - 1} H\left( {{\text{x}},{\text{y}},{\text{t}}} \right))^{n} } \right\}} \right] + {\text{ BT}}\left[ {{ }\left\{ {D_{xyy} { }(T^{ - 1} H\left( {{\text{x}},{\text{y}},{\text{t}}} \right))^{n} } \right\}} \right] = 0. \\ \end{aligned}$$

Multiply Eq. (4) by $q^{ - \alpha } \left( s \right)$ we get

5 $$\begin{aligned} & H\left( {x,y,s} \right) - \varphi \left( s \right)q^{ - 1} \left( s \right) H\left( {x,y,0} \right) + { }q^{ - \alpha } \left( s \right){\text{T}}\left[ {{ }\left\{ {D_{x} (T^{ - 1} H\left( {{\text{x}},{\text{y}},{\text{t}}} \right))^{n} } \right\}} \right] \\ & \quad + q^{ - \alpha } \left( s \right){\text{BT}}\left[ {{ }\left\{ {{ }D_{x}^{3} (T^{ - 1} H\left( {{\text{x}},{\text{y}},{\text{t}}} \right))^{n} } \right\}} \right] + q^{ - \alpha } \left( s \right){\text{ BT}}\left[ {{ }\left\{ {D_{xyy} { }(T^{ - 1} H\left( {{\text{x}},{\text{y}},{\text{t}}} \right))^{n} } \right\}} \right] = 0. \\ \end{aligned}$$

*Step 2* The transformed function can be written using the following RPSM expansion:

6 $$H\left( {x,y,s} \right) = \varphi \left( s \right)\mathop \sum \limits_{n = 1}^{\infty } \frac{{f_{n} \left( {x,y} \right)}}{{q^{n\alpha + 1} \left( s \right)}}.$$

Equation (6) is a Mittag–Leffler fractional series, specifically designed for Caputo fractional derivatives. This equation generalizes Taylor series and is a natural fit with the memory structure of fractional operators.

The kth-truncated series (6) take the form:

7 $$H_{k} \left( {x,y,s} \right) = \varphi \left( s \right)\mathop \sum \limits_{n = 1}^{k} \frac{{f_{n} \left( {x,y} \right)}}{{q^{n\alpha + 1} \left( s \right)}} = \varphi \left( s \right)\frac{{ f_{0} \left( {x,y} \right)}}{q\left( s \right)} + \varphi \left( s \right) \mathop \sum \limits_{n = 1}^{k} \frac{{f_{n} \left( {x,y} \right)}}{{q^{n\alpha + 1} \left( s \right)}}.$$

The new general integral residual function defines as:

8 $$\begin{aligned} TRes\left( {x,y ,s} \right) & = {\text{H}}\left( {{\text{x}},{\text{y}},{\text{s}}} \right) - \varphi \left( s \right)q^{ - 1} \left( s \right){\text{H}}\left( {{\text{x}},{\text{y}},0} \right) + { }q^{ - \alpha } \left( s \right){\text{T}}\left[ {{ }\left\{ {D_{x} (T^{ - 1} H\left( {{\text{x}},{\text{y}},{\text{t}}} \right))^{n} } \right\}} \right] \\ & \quad + q^{ - \alpha } \left( s \right){\text{BT}}\left[ {{ }\left\{ {{ }D_{x}^{3} (T^{ - 1} H\left( {{\text{x}},{\text{y}},{\text{t}}} \right))^{n} } \right\}} \right] + q^{ - \alpha } \left( s \right){\text{ BT}}\left[ {{ }\left\{ {D_{xyy} { }(T^{ - 1} H\left( {{\text{x}},{\text{y}},{\text{t}}} \right))^{n} } \right\}} \right]. \\ \end{aligned}$$

Equation (8) yields a residual function $Res\left( {x,y ,s} \right)$ that guarantees a gradually decreasing residual value, as a fixed-point iteration.

The kth-new general integral residual function defines as:

9 $$\begin{aligned} TRes_{k} \left( {x,y,s} \right) & = H_{k} \left( {{\text{x}},{\text{y}},{\text{s}}} \right) - \varphi \left( s \right)q^{ - 1} \left( s \right)H_{k} \left( {{\text{x}},{\text{y}},0} \right) + { }q^{ - \alpha } \left( s \right){\text{T}}\left[ {{ }\left\{ {D_{x} T^{ - 1} \{ H_{k}^{n} \left( {{\text{x}},{\text{y}},{\text{t}}} \right)} \right\}} \right] \\ & \quad + q^{ - \alpha } \left( s \right){\text{BT}}\left[ {{ }\left\{ {{ }D_{x}^{3} T^{ - 1} \{ H_{k}^{n} \left( {{\text{x}},{\text{y}},{\text{t}}} \right)} \right\}} \right] + q^{ - \alpha } \left( s \right){\text{ BT}}\left[ {{ }\left\{ {D_{xyy} T^{ - 1} \{ { }H_{k}^{n} \left( {{\text{x}},{\text{y}},{\text{t}}} \right)} \right\}} \right]. \\ \end{aligned}$$

Substituting Eq. (7) into Eq. (9), and multiplying the resulting equation by ${q}^{k\alpha +1}\left(s\right)$, we get

10 $$\begin{aligned} & q^{k\alpha + 1} \left( s \right)TRes_{k} \left( {x,y,s} \right) = \varphi \left( s \right)q^{k\alpha + 1} \left( s \right)\sum\nolimits_{n = 1}^{k} {\frac{{f_{n} \left( {x,y} \right)}}{{q^{n\alpha + 1} \left( s \right)}} + q^{k\alpha + 1} \left( s \right){ }q^{ - \alpha } \left( s \right)} \\ & \quad \quad {\text{T}}\left[ {{ }\left\{ {D_{x} T^{ - 1} (\varphi \left( s \right)\frac{{ f_{0} \left( {x,y} \right)}}{q\left( s \right)} + \varphi \left( s \right) \mathop \sum \limits_{n = 1}^{k} \frac{{f_{n} \left( {x,y} \right)}}{{q^{n\alpha + 1} \left( s \right)}})^{n} } \right\}} \right] \\ & \quad + q^{k\alpha + 1} \left( s \right)q^{ - \alpha } \left( s \right){\text{BT}}\left[ {{ }\left\{ {{ }D_{x}^{3} T^{ - 1} (\varphi \left( s \right)\frac{{ f_{0} \left( {x,y} \right)}}{q\left( s \right)} + \varphi \left( s \right) \mathop \sum \limits_{n = 1}^{k} \frac{{f_{n} \left( {x,y} \right)}}{{q^{n\alpha + 1} \left( s \right)}})^{n} } \right\}} \right] \\ & \quad + q^{k\alpha + 1} \left( s \right)q^{ - \alpha } \left( s \right){\text{ BT}}\left[ {{ }\left\{ {D_{xyy} T^{ - 1} (\varphi \left( s \right)\frac{{ f_{0} \left( {x,y} \right)}}{q\left( s \right)} + \varphi \left( s \right) \mathop \sum \limits_{n = 1}^{k} \frac{{f_{n} \left( {x,y} \right)}}{{q^{n\alpha + 1} \left( s \right)}})^{n} { }} \right\}} \right]. \\ \end{aligned}$$

We can find the coefficient function $f_{n} \left( {x,y} \right)$ by solving the following system:

$$\mathop {\lim }\limits_{q\left( s \right) \to \infty } q^{k\alpha + 1} \left( s \right)TRes_{k} \left( s \right) = 0:{\text{k}} = {1},{2},{3}$$

## The error norms

To investigate the accuracy and applicability of GIRPSM, we presented numerical outcomes through an error norms which defined in^48^ as follows:

$$\begin{aligned} & L_{\infty } = \mathop {\max }\limits_{0 \le j \le M} \left( {E_{j} } \right), \\ & L_{2} = \sqrt {h\mathop \sum \limits_{j = 0}^{M} (E_{j} )^{2} ,} \\ & where\;E_{j} = \left| {u_{i} \left( {Exact} \right) - u_{i} \left( {Approximate} \right)} \right| \\ \end{aligned}$$

$$L_{2} = \sqrt {h\mathop \sum \limits_{j = 0}^{M} (E_{j} )^{2} ,}$$

$$where E_{j} = \left| {u_{i} \left( {Exact} \right) - u_{i} \left( {Approximate} \right)} \right|$$.

## Numerical examples

### Example 1

Examine the fractional Zakharov–Kuznetsov equation that follows.

11 $$D_{t}^{\alpha } H + H_{x }^{2} + \frac{1}{8}H_{xxx}^{2} + \frac{1}{8}H_{xyy}^{2} = 0,$$

with the initial condition:

12 $$H\left( {x,y,0} \right) = \frac{4}{3}\rho \;sinh^{2} \left( {x + y} \right),$$

where $\rho$ is a constant representing wave speed.

The exact solution at $\alpha = 1$ is

13 $$H\left( {x,y,t} \right) = \frac{4}{3}\rho sinh^{2} \left( {x + y - \rho t} \right).$$

We will start the present method by taking the initial value:

14 $$f_{0} \left( {x,y} \right) = H\left( {x,0} \right) = \frac{4}{3}\rho \;sinh^{2} \left( {x + y} \right).$$

Substitute k = 1 into Eq. (11) to create the coefficient function $f_{1} \left( {x,y} \right)$ as:

15 $$\mathop {\lim }\limits_{q\left( s \right) \to \infty } q^{\alpha + 1} \left( s \right)TRes_{1} \left( s \right) = 0.$$

Then, we get

16 $$f_{1} \left( {x,y} \right) = - \left\{ {\frac{\partial }{\partial x}f_{0}^{2} + \frac{1}{8} \frac{{\partial^{3} }}{{\partial x^{3} }}f_{0}^{2} + \frac{1}{8} \frac{{\partial^{3} }}{{\partial x\partial y^{2} }}f_{0}^{2} } \right\},$$

17 $$f_{1} \left( {x,y} \right) = \frac{ - 8}{3}\rho^{2} \sinh \left( {4\left( {x + y} \right)} \right).$$

Substitute k = 2 into Eq. (11) to create the coefficient function $f_{2} \left( {x,y} \right)$ as:

18 $$\mathop {\lim }\limits_{q\left( s \right) \to \infty } q^{2\alpha + 1} \left( s \right)TRes_{2} \left( s \right) = 0.$$

Then, we get

19 $$f_{2} \left( {x,y} \right) = - 2\left\{ { D_{x} \left( {f_{0} f_{1} } \right) + \frac{1}{8}D_{xxx} \left( {f_{0} f_{1} } \right) + \frac{1}{8}D_{xyy} \left( {f_{0} f_{1} } \right) } \right\},$$

20 $$f_{2} \left( {x,y} \right) = \frac{64}{9} \rho^{3} (15\cosh \left( {6\left( {x + y} \right)} \right) - 10{\text{cosh}}(4\left( {x + y} \right) + \cosh \left( {2\left( {x + y} \right)} \right).$$

Substitute k = 3 into Eq. (11) to create the coefficient function $f_{3} \left( {x,y} \right)$ as:

21 $$\mathop {\lim }\limits_{q\left( s \right) \to \infty } q^{3\alpha + 1} \left( s \right)TRes_{2} \left( s \right) = 0.$$

Then, we get

22 $$f_{3} \left( {x,y} \right) = - 2\left\{ { D_{x} \left( {f_{0} f_{2} } \right) + \frac{1}{8}D_{xxx} \left( {f_{0} f_{2} } \right) + \frac{1}{8}D_{xyy} \left( {f_{0} f_{2} } \right)} \right\} - \{ D_{x} ( f_{0} )^{2} + \frac{1}{8} D_{xxx} ( f_{0} )^{2} + \frac{1}{8} D_{xyy} {(} f_{0} )^{2} {\text{\} }}\frac{{{\Gamma }\left( {2\alpha + 1} \right)}}{{\left( {{\Gamma }\left( {\alpha + 1} \right)} \right)^{2} }},$$

23 $$\begin{aligned} f_{3} \left( {x,y} \right) & = - \frac{1024}{9} \rho^{4} \left\{ {85\sinh \left( {8\left( {x + y} \right)} \right) - 100\sinh \left( {6\left( {x + y} \right)} \right) + 30{\text{sinh}}\left( {4\left( {x + y} \right)} \right)} \right. \\ & \quad \left. { - 2{\text{sinh}}\left( {2\left( {x + y} \right)} \right) + \left( {\frac{4352}{9} sinh\left( {8\left( {x + y} \right)} \right) \frac{{{\Gamma }\left( {2\alpha + 1} \right)}}{{\left( {{\Gamma }\left( {\alpha + 1} \right)} \right)^{2} }}} \right)} \right\}. \\ \end{aligned}$$

The approximate solution with GIRPS take the form:

24 $$\begin{aligned} H\left( {x,y,t} \right) & = \frac{4}{3} \rho sinh^{2} \left( {x + y} \right) - \frac{8}{3}\rho^{2} \sinh \left( {4\left( {x + y} \right)} \right)\frac{{t^{\alpha } }}{{{\Gamma }\left( {\alpha + 1} \right)}} + \frac{64}{9} \rho^{3} (15\cosh \left( {6\left( {x + y} \right)} \right) \\ & \quad - 10{\text{cosh}}\left( {4\left( {x + y} \right) + {\text{cosh}}\left( {2\left( {x + y} \right)} \right) } \right) { }\frac{{t^{2\alpha } }}{{{\Gamma }\left( {2\alpha + 1} \right)}} - \frac{1024}{9}\rho^{4} \left\{ {(85\sinh \left( {8\left( {x + y} \right)} \right) - 100\sinh \left( {6\left( {x + y} \right)} \right)} \right. \\ & \quad \left. { + 30{\text{sinh}}\left( {4\left( {x + y} \right)} \right) - 2{\text{sinh}}\left( {2\left( {x + y} \right)} \right) + \frac{4352}{9} sinh\left( {8\left( {x + y} \right)} \right) \frac{{{\Gamma }\left( {2\alpha + 1} \right)}}{{\left( {{\Gamma }\left( {\alpha + 1} \right)} \right)^{2} }}} \right\} \frac{{t^{3\alpha } }}{{{\Gamma }\left( {3\alpha + 1} \right)}} + \ldots \\ \end{aligned}$$

### Example 2

Consider the fractional Zakharov -Kuznetsov Eq. (2) at n = 3, B = 2.

25 $$D_{t}^{\alpha } H + H_{x}^{3} + 2H_{xxx}^{3} + 2H_{xyy}^{3} = 0,$$

with the initial condition

26 $$H\left( {x,y,0} \right) = \frac{3}{2}\rho \sinh \left( {\frac{1}{6}\left( {x + y} \right)} \right).$$

In the classical case, the exact solution is

27 $$H\left( {x,y,t} \right) = \frac{3}{2}\rho \sinh \left( {\frac{ 1}{6}\left( {x + y - \rho t} \right)} \right).$$

Repeat the same steps as in the first example1 and start with the initial value:

28 $$f_{0} \left( {x,y} \right) = u\left( {x,y,0} \right) = \frac{3}{2}\rho \sinh \left( {\frac{1}{6}\left( {x + y} \right)} \right).$$

The coefficient functions take the form:

29 $$f_{1} \left( {x,y} \right) = \frac{ - 3}{{32}} \rho^{3} (9{\text{cosh}}\left( {\frac{1}{2}\left( {x + y} \right)} \right) - 5 {\text{cosh}}\left( {\frac{1}{6}\left( {x + y} \right)} \right).$$

30 $$f_{2} \left( {x,y} \right) = \frac{3}{1024} \rho^{5} \left( {26543205\sinh \left( {\frac{{5\left( {x + y} \right)}}{6}} \right) + 40698477sinh\left( {\frac{{\left( {x + y} \right)}}{2}} \right) + 8257550{\text{sinh}}\left( {\frac{{\left( {x + y} \right)}}{6}} \right)} \right).$$

31 $$\begin{aligned} f_{3} \left( {x,y} \right) & = \left\{ { - \frac{9}{16384} \rho^{7} \left[ {1796090205\cosh \left( {\frac{{7\left( {x + y} \right)}}{6}} \right) - 2657238465\cosh \left( {\frac{{5\left( {x + y} \right)}}{6}} \right)} \right.} \right. \\ & \quad + 1045779381\cosh \left( {\frac{{\left( {x + y} \right)}}{2}} \right) - \left. {109118545{\text{cosh}}\left( {\frac{{\left( {x + y} \right)}}{6}} \right)} \right] \\ & \quad - \frac{9}{16384} \rho^{7} \left[ {16443\cosh \left( {\frac{{7\left( {x + y} \right)}}{6}} \right) - 14535\cosh \left( {\frac{{5\left( {x + y} \right)}}{6}} \right)} \right. \\ & \quad \left. {\left. { + 675\cosh \left( {\frac{{\left( {x + y} \right)}}{2}} \right) + 1385{\text{cosh}}\left( {\frac{{\left( {x + y} \right)}}{6}} \right)} \right] \frac{{{\Gamma }\left( {2\alpha + 1} \right)}}{{\left( {{\Gamma }\left( {\alpha + 1} \right)} \right)^{2} }}} \right\}. \\ \end{aligned}$$

The approximate solution with GIRPSM take the form:

32 $$\begin{aligned} H\left( {x,y,t} \right) & = \frac{3}{2} \rho sinh\left( {\frac{1}{6}\left( {x + y} \right)} \right) - \left\{ {\frac{3}{32} \rho^{3} (9cosh\left( {\frac{1}{2}\left( {x + y} \right)} \right) - 5 cosh\left( {\frac{1}{6}\left( {x + y} \right)} \right)} \right\}\frac{{t^{\alpha } }}{{{\Gamma }\left( {\alpha + 1} \right)}} \\ & \quad + \left\{ {\frac{3}{1024} \rho^{5} (26543205\sinh \left( {\frac{{5\left( {x + y} \right)}}{6}} \right) + 40698477sinh\left( {\frac{{\left( {x + y} \right)}}{2}} \right) + 8257550{\text{sinh}}\left( {\frac{{\left( {x + y} \right)}}{6}} \right)} \right\}\frac{{t^{2\alpha } }}{{{\Gamma }\left( {2\alpha + 1} \right)}} \\ & \quad - \left\{ {\frac{9}{16384} \rho^{7} \left[ {1796090205\cosh \left( {\frac{{7\left( {x + y} \right)}}{6}} \right) - 2657238465\cosh \left( {\frac{{5\left( {x + y} \right)}}{6}} \right) + 1045779381\cosh \left( {\frac{{\left( {x + y} \right)}}{2}} \right)} \right.} \right. \\ & \quad \left. { - 109118545{\text{cosh}}\left( {\frac{{\left( {x + y} \right)}}{6}} \right)} \right] + \frac{9}{16384} \rho^{7} \left[ {16443\cosh \left( {\frac{{7\left( {x + y} \right)}}{6}} \right) - 14535\cosh \left( {\frac{{5\left( {x + y} \right)}}{6}} \right) + 675\cosh \left( {\frac{{\left( {x + y} \right)}}{2}} \right)} \right. \\ & \quad \left. {\left. { + 1385{\text{cosh}}\left( {\frac{{\left( {x + y} \right)}}{6}} \right)} \right]\frac{{{\Gamma }\left( {2\alpha + 1} \right)}}{{\left( {{\Gamma }\left( {\alpha + 1} \right)} \right)^{2} }}} \right\}\frac{{t^{3\alpha } }}{{{\Gamma }\left( {3\alpha + 1} \right)}} + \ldots \\ \end{aligned}$$

## Discussions

**Fig. 1:**
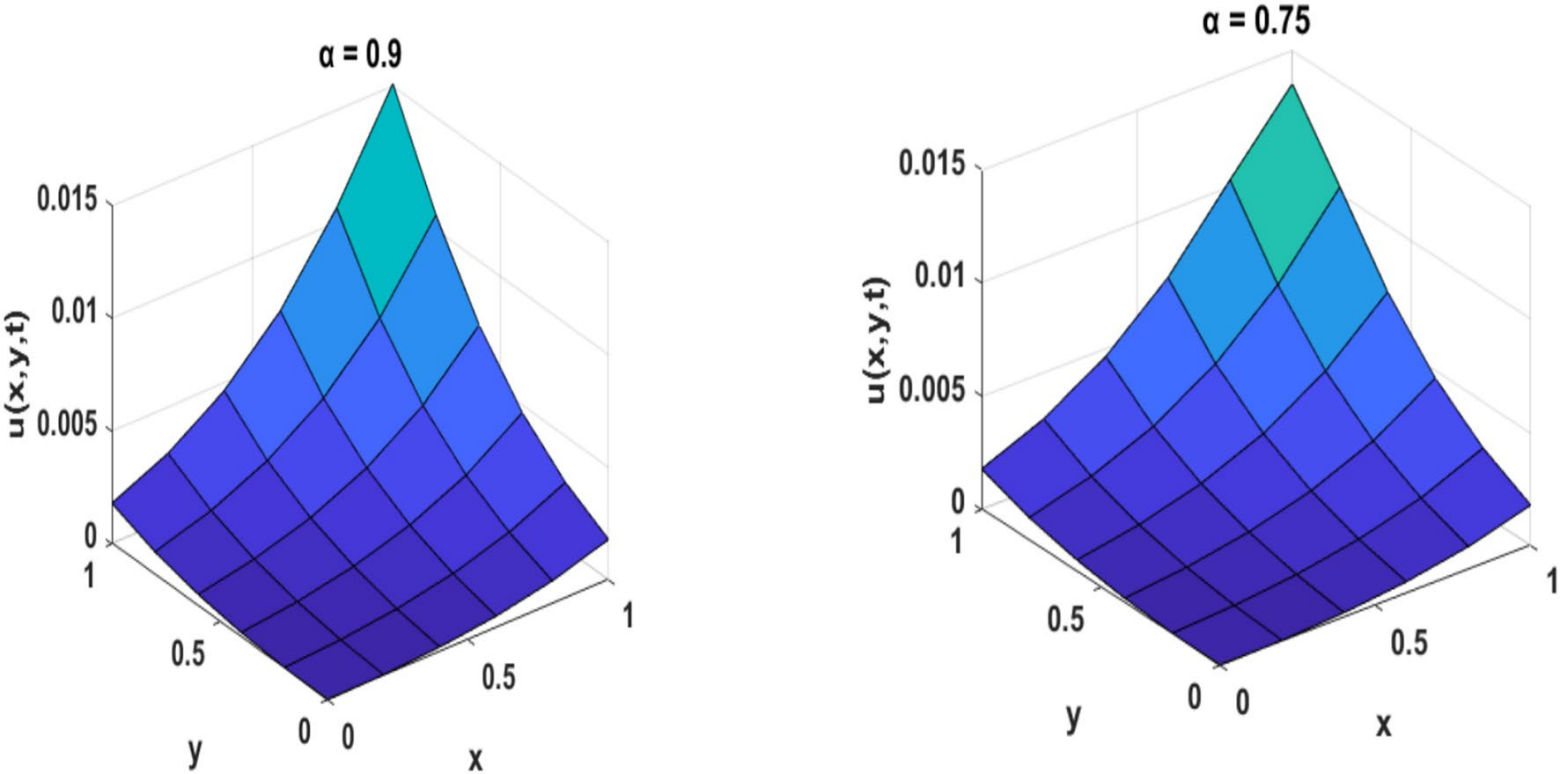
Surface plots representing numerical solutions for example 1 at $\rho = 0.001$, t = 0.5

**Fig. 2:**
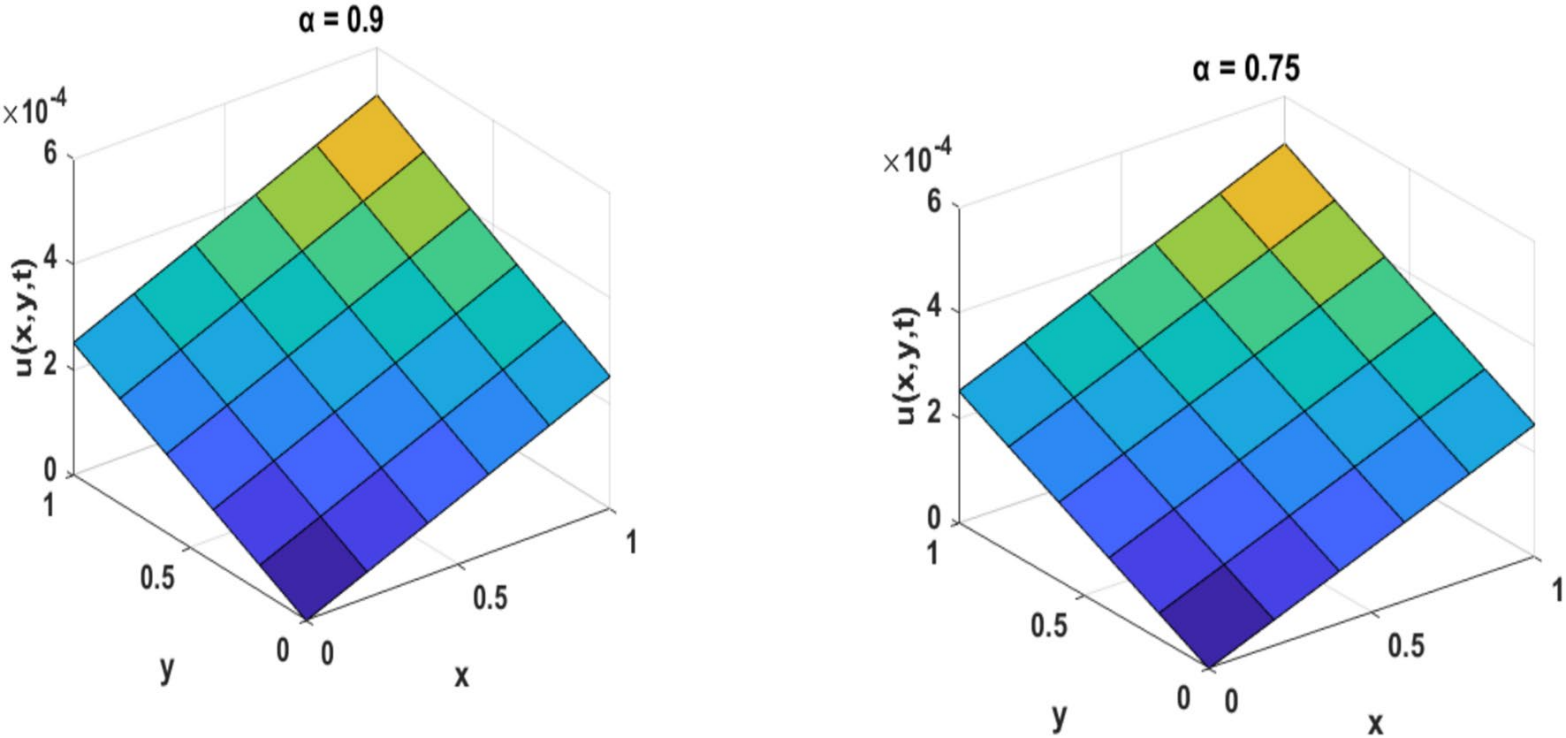
Surface plots representing numerical solutions for example 2 at $\rho =0.001$, t = 0.5

**Fig. 3:**
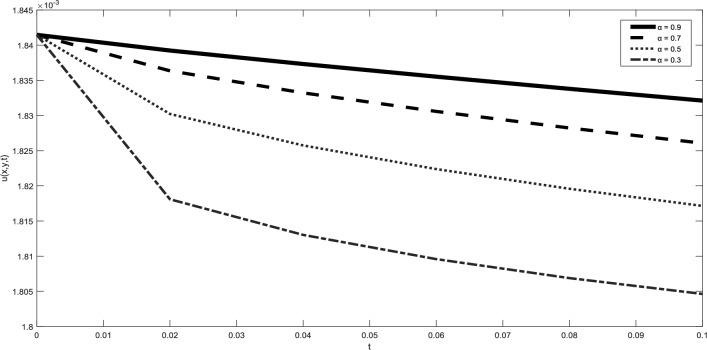
2D plots representing numerical solutions for example 1 at $\rho = 0.001$, x = 0.5 and y = 0.5

**Fig. 4:**
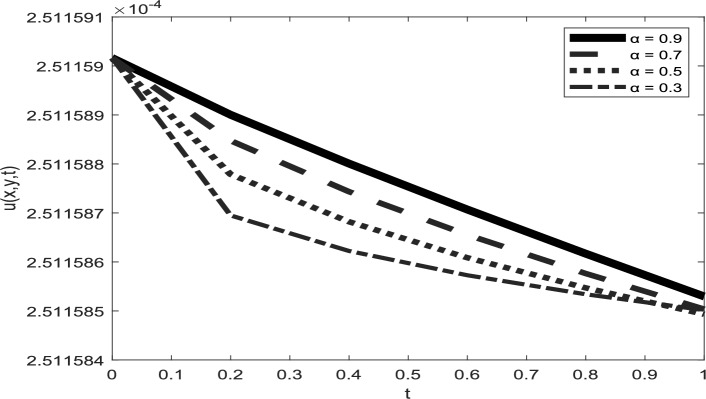
2D plots of the numerical solutions for example 1 at $\rho =0.001$, x = 0.5 and y = 0.5

**Fig. 5:**
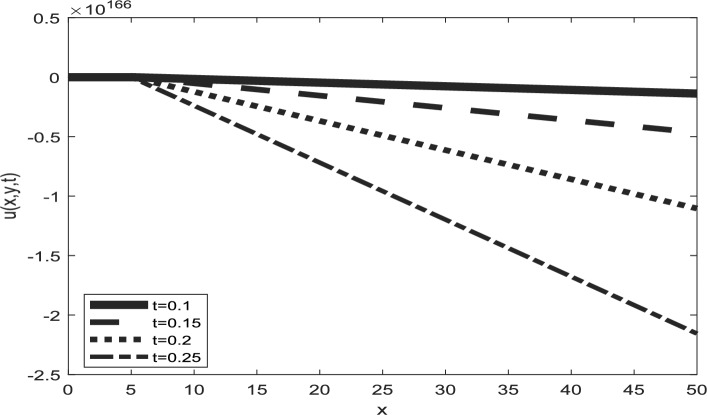
2D plots representing numerical solutions for example 1 at $\rho = 0.001$, α = 1 and y = 1.

**Fig. 6:**
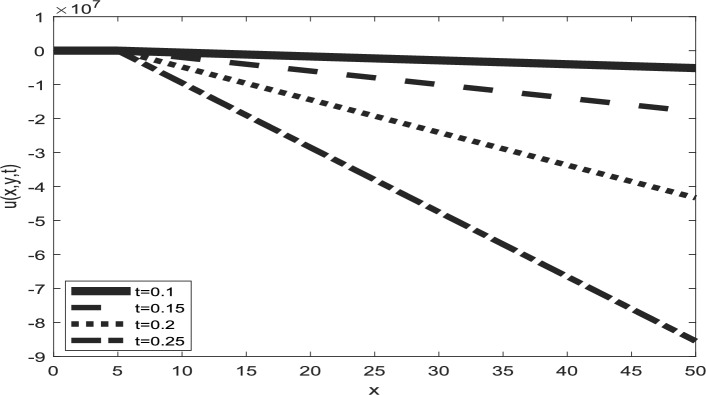
2D plots representing numerical solutions for example 2 at $\rho =0.001$, α = 1 and y = 1.

Table 1 presents a comparison between GIRPSM with q-HAShTM, VIM, FNDM, and q-HATM for example 1. Table 2 represents the absolute errors between the approximate and exact solutions across different values of the fractional order α, providing important insights into the performance of the GIRPSM for example 1. Table 3 shows the comparison between GIRPSM with q-HAShTM, FNDM, and q-HATM for example 2. The error magnitude generally decreases with increasing number of terms in the power series, confirming the method’s convergence behavior. Table 4 shows the absolute errors between the approximate and exact solutions across different values of the fractional order α, providing important insights into the performance of the GIRPSM for example 2. Tables 5 and 6 presents the error norms as a benchmark for the reliability of the used method and the q-HAShTM^37^ for the first and second examples. the q-HAShTM method is similar in its results to the other methods FNDM, and q-HATM. The tables highlight the superiority of the GIRPSM method over other methods, proving its accuracy and reliability. Figures 1 and 2 show the behavior of 3D solutions for example 1 and example 2. Figures 3 and 4 show the behavior of 2D solutions for example 1 and example at different α values. Figures 5 and 6 show the behavior of 2D solutions for example 1 and example 2 at different t values. From the previous results we find that GIRPSM has local accuracy and stability, but over long timescales, its reliability depends on the convergence of the series and its residual decay. This method does not automatically guarantee global stability without careful time-step management or control of series truncation. However, using adaptive techniques, GIRPSM can be reliably used over extended timescales in many practical problems. This series converges for all finite values of *t* but is slower for larger values of *t* or small α, as is the case with most series-based methods, especially for nonlinear problems. This method constructs the solution as a fractional power series with residual corrections, which converges locally near the starting point *t* = 0.

**Table 1:** Comparison between GIRPSM with the q-HAShTM, VIM, FNDM, q-HATM for example1 at t = 1, $\alpha = 1$, $\rho = 0.001$.

x	y	Numerical solution				
		q-HAShTM^36^	VIM^32^	FNDM^34^	q-HATM^34^	GIRPSM
0.02	0.02	3.00 $*{10}^{-7}$	7.90 $*{10}^{-6}$	3.01 $*{10}^{-7}$	3.01 $*{10}^{-7}$	3.00 $*{10}^{-7}$
	0.04	4.65 $*{10}^{-7}$	1.19 $*{10}^{-5}$	4.65 $*{10}^{-7}$	4.65 $*{10}^{-7}$	4.63 $*{10}^{-7}$
	0.06	6.34 $*{10}^{-7}$	1.59 $*{10}^{-5}$	6.34 $*{10}^{-7}$	6.34 $*{10}^{-7}$	6.29 $*{10}^{-7}$
	0.08	8.10 $*{10}^{-7}$	2.00 $*{10}^{-5}$	8.10 $*{10}^{-7}$	8.10 $*{10}^{-7}$	8.00 $*{10}^{-7}$
	0.1	9.96 $*{10}^{-7}$	2.41 $*{10}^{-5}$	9.96 $*{10}^{-7}$	9.96 $*{10}^{-7}$	9.77 $*{10}^{-7}$
0.06	0.02	6.34 $*{10}^{-7}$	1.59 ${*10}^{-5}$	6.34 $*{10}^{-7}$	6.34 $*{10}^{-7}$	6.29 $*{10}^{-7}$
	0.04	8.10 $*{10}^{-7}$	2.00 $*{10}^{-5}$	8.10 $*{10}^{-7}$	8.10 $*{10}^{-7}$	8.00 $*{10}^{-7}$
	0.06	9.96 $*{10}^{-7}$	2.41 ${*10}^{-5}$	9.96 $*{10}^{-7}$	9.96 $*{10}^{-7}$	9.77 $*{10}^{-7}$
	0.08	1.19 $*{10}^{-6}$	2.84 $*{10}^{-5}$	1.19 $*{10}^{-6}$	1.19 $*{10}^{-6}$	1.16 $*{10}^{-6}$
	0.1	1.40 $*{10}^{-6}$	3.27 $*{10}^{-5}$	1.40 $*{10}^{-6}$	1.40 $*{10}^{-6}$	1.35 $*{10}^{-6}$
0.1	0.02	9.96 $*{10}^{-7}$	2.41 $*{10}^{-5}$	9.96 $*{10}^{-6}$	9.96 $*{10}^{-7}$	9.77 $*{10}^{-7}$
	0.04	1.19 $*{10}^{-6}$	2.84 $*{10}^{-5}$	1.19 $*{10}^{-6}$	1.19 $*{10}^{-6}$	1.16 $*{10}^{-6}$
	0.06	1.40 $*{10}^{-6}$	3.27 $*{10}^{-5}$	1.40 $*{10}^{-6}$	1.40 $*{10}^{-6}$	1.35 $*{10}^{-6}$
	0.08	1.62 $*{10}^{-6}$	3.72 $*{10}^{-5}$	1.63 $*{10}^{-6}$	1.63 $*{10}^{-6}$	1.55 $*{10}^{-6}$
	0.1	1.87 $*{10}^{-6}$	418 $*{10}^{-5}$	1.87 $*{10}^{-6}$	1.87 $*{10}^{-6}$	1.77 $*{10}^{-6}$

**Table 2:** Numerical outcome to example 1.

x	y	t	Exact	GIRPSM		
				α = 0.75	α = 0.9	α = 1
0.1	0.1	0.1	5.399 $*{10}^{-5}$	5.359 $*{10}^{-5}$	5.373 $*{10}^{-5}$	5.381 $*{10}^{-5}$
		0.25	5.391 $*{10}^{-5}$	5.314 $*{10}^{-5}$	5.334 $*{10}^{-5}$	5.345 $*{10}^{-5}$
		0.5	5.377 $*{10}^{-5}$	5.254 $*{10}^{-5}$	5.274 $*{10}^{-5}$	5.287 $*{10}^{-5}$
		0.75	5.363 $*{10}^{-5}$	5.202 $*{10}^{-5}$	5.218 $*{10}^{-5}$	5.230 $*{10}^{-5}$
		1	5.350 $*{10}^{-5}$	5.154 $*{10}^{-5}$	5.164 $*{10}^{-5}$	5.173 $*{10}^{-5}$
0.5	0.5	0.1	0.001840	0.001827	0.0018321	0.001834
		0.25	0.001840	0.001815	0.0018206	0.001823
		0.5	0.001839	0.001798	0.0018037	0.001807
		0.75	0.001837	0.001784	0.0017885	0.001791
		1	0.001836	0.001771	0.0017742	0.001776
1	1	0.1	0.017535	0.016873	0.0170776	0.017176
		0.25	0.017529	0.016028	0.0165129	0.016692
		0.5	0.017520	0.013501	0.0150977	0.015657
		0.75	0.017511	0.008914	0.0123300	0.013710
		1	0.017502	0.001843	0.0075050	0.010127

**Table 3:** Comparison between errors of q-HAShTM, FNDM and q-HATM with GIRPSM for example 2 at $\alpha = 1$, $\rho = 0.001$.

x = y	t	Numerical solution			
		GIRPSM	q-HATM^34^	FNDM^34^	q-HAShTM^36^
0.02	2*${10}^{-2}$	4.9926*${10}^{-9}$	4.9926*${10}^{-9}$	4.9926*${10}^{-9}$	4.9926*${10}^{-9}$
	4*${10}^{-2}$	9.9852*${10}^{-9}$	9.9852*${10}^{-9}$	9.9852*${10}^{-9}$	9.9852*${10}^{-9}$
	6*${10}^{-2}$	1.4977*${10}^{-8}$	1.4979*${10}^{-8}$	1.4979*${10}^{-8}$	1.4977*${10}^{-8}$
	8*${10}^{-2}$	1.9970*${10}^{-8}$	1.9970*${10}^{-8}$	1.9970*${10}^{-8}$	1.9970*${10}^{-8}$
	1*${10}^{-1}$	2.4963*${10}^{-8}$	2.4963*${10}^{-8}$	2.4963*${10}^{-8}$	2.4963*${10}^{-8}$
0.06	2*${10}^{-2}$	4.9934*${10}^{-9}$	4.9963*${10}^{-9}$	4.9963*${10}^{-9}$	4.9934*${10}^{-9}$
	4*${10}^{-2}$	9.9869*${10}^{-9}$	9.9869*${10}^{-9}$	9.9869*${10}^{-9}$	9.9869*${10}^{-9}$
	6*${10}^{-2}$	1.4980*${10}^{-8}$	1.4980*${10}^{-8}$	1.4980*${10}^{-8}$	1.4980*${10}^{-8}$
	8*${10}^{-2}$	1.9973*${10}^{-8}$	1.9974*${10}^{-8}$	1.9974*${10}^{-8}$	1.9973*${10}^{-8}$
	1*${10}^{-1}$	2.4967*${10}^{-8}$	2.4967*${10}^{-8}$	2.4967*${10}^{-8}$	2.4967*${10}^{-8}$
0.1	2*${10}^{-2}$	4.9951*${10}^{-9}$	4.9952*${10}^{-9}$	4.9952*${10}^{-9}$	4.9951*${10}^{-9}$
	4*${10}^{-2}$	9.9903*${10}^{-9}$	9.9904*${10}^{-9}$	9.9904*${10}^{-9}$	9.9904*${10}^{-9}$
	6*${10}^{-2}$	1.4985*${10}^{-8}$	1.4986*${10}^{-8}$	1.4986*${10}^{-8}$	1.4985*${10}^{-8}$
	8*${10}^{-2}$	1.9980*${10}^{-8}$	1.9981*${10}^{-8}$	1.9981*${10}^{-8}$	1.9980*${10}^{-8}$
	1*${10}^{-1}$	2.4975*${10}^{-8}$	2.4976*${10}^{-8}$	2.4976*${10}^{-8}$	2.4976*${10}^{-8}$

**Table 4:** Numerical outcome to example 2.

x/y	t	Exact	GIRPSM		
			α = 0.75	α = 0.9	α = 1
0.1	0.1	4.9984245*${10}^{-5}$	5.0009186*${10}^{-5}$	5.0009210*${10}^{-5}$	5.0009221*${10}^{-5}$
	0.25	4.9946725*${10}^{-5}$	5.0009114*${10}^{-5}$	5.0009146*${10}^{-5}$	5.0009165*${10}^{-5}$
	0.5	4.9884190*${10}^{-5}$	5.0009015*${10}^{-5}$	5.0009048*${10}^{-5}$	5.0009070*${10}^{-5}$
	0.75	4.9821655*${10}^{-5}$	5.0008928*${10}^{-5}$	5.0008956*${10}^{-5}$	5.0008976*${10}^{-5}$
	1	4.9759121*${10}^{-5}$	5.0008848*${10}^{-5}$	5.0008866*${10}^{-5}$	5.0008881*${10}^{-5}$
0.5	0.1	0.0002511336	0.0002511589	0.00025115895	0.00025115896
	0.25	0.0002510956	0.0002511588	0.00025115887	0.00025115889
	0.5	0.0002510322	0.0002511587	0.00025115875	0.00025115877
	0.75	0.0002509689	0.0002511586	0.00025115863	0.00025115866
	1	0.0002509055	0.0002511585	0.00025115852	0.00025115854
1	0.1	0.0005092844	0.0005093106	0.00050931073	0.00050931075
	0.25	0.0005092448	0.0005093105	0.00050931059	0.00050931063
	0.5	0.0005091788	0.0005093103	0.00050931039	0.00050931044
	0.75	0.0005091128	0.0005093101	0.00050931021	0.00050931024
	1	0.0005090468	0.0005093100	0.00050931003	0.00050931006

**Table 5:** Error norms comparison between GIRPSM with the q-HAShTM for example at $t=0.05$.

	GIRPSM		q-AShTM^37^	
$x/y$	${L}_{2}$	${L}_{\infty }$	${L}_{2}$	${L}_{\infty }$
0.1	1.01632e−09	9.09023e−08	2.17167e−08	9.71201e−088
0.25	4.51934e−09	4.04222e−07	1.16038e−07	5.18937e−07
0.5	3.77075e−08	3.37266e−06	1.15516e−06	5.16601e−06
0.75	2.87581e−07	2.57220e−05	9.38855e−06	4.19869e−05
1	2.09250e−06	1.87159e−04	7.04331e−05	3.14986e−04

**Table 6:** Error norms comparison between GIRPSM with q-HAShTM for example 2 at $t=0.05$.

$x/y$	GIRPSM		q-HAShTM^36^	
	${L}_{2}$	${L}_{\infty }$	${L}_{2}$	${L}_{\infty }$
0.1	1.39620e−10	1.24880e−08	2.79240e−09	1.24880e−08
0.25	1.40016e−10	1.25234e−08	2.80032e−09	1.25234e−08
0.5	1.41434e−10	1.26502e−08	2.82867e−09	1.26502e−08
0.75	1.43804e−10	1.28622e−08	2.87608e−09	1.28622e−08
1	1.47140e−10	1.31606e−08	2.94278e−09	1.31606e−08

## Conclusion

In this paper, GIRPSM was successfully applied to obtain approximate analytical solutions to the FZKE. The performance of GIRPSM is highly sensitive to the choice of initial approximation and fractional order and may not be as robust in boundary value problems with complex geometries or sharp transitions. Compared to other numerical methods, GIRPSM is a promising semi-analytical technique for solving fractional differential equations. The order of convergence of GIRPSM is typically exponential, especially for problems with smooth analytic solutions. This makes it a highly efficient method compared to traditional series or perturbation techniques, which often exhibit only linear or polynomial convergence. Future work could explore hybrid approaches that combine GIRPSM with numerical correction techniques to overcome convergence and domain limitations. Future work will extend this method to more complex non-homogeneous systems to enhance its versatility. By explicitly defining the residual and integral correction, other researchers can now apply GIRPSM directly to their nonlinear fractional models.

## Data Availability

Raw data supporting the findings of this study are available upon request from the corresponding author.
